# ROCK2-Specific Inhibitor KD025 Suppresses Adipocyte Differentiation by Inhibiting Casein Kinase 2

**DOI:** 10.3390/molecules26164747

**Published:** 2021-08-05

**Authors:** Nhu Nguyen Quynh Tran, Kwang-Hoon Chun

**Affiliations:** Gachon Institute of Pharmaceutical Sciences, College of Pharmacy, Gachon University, Incheon 21936, Korea; quynhnhutrannguyen10@gmail.com

**Keywords:** KD025, belumosudil, ROCK2, casein kinase, CK2, adipocyte differentiation

## Abstract

KD025, a ROCK2 isoform-specific inhibitor, has an anti-adipogenic activity which is not mediated by ROCK2 inhibition. To identify the target, we searched binding targets of KD025 by using the KINOMEscan^TM^ screening platform, and we identified casein kinase 2 (CK2) as a novel target. KD025 showed comparable binding affinity to CK2α (*K*_d_ = 128 nM). By contrast, CK2 inhibitor CX-4945 and ROCK inhibitor fasudil did not show such cross-reactivity. In addition, KD025 effectively inhibited CK2 at a nanomolar concentration (IC_50_ = 50 nM). We examined if the inhibitory effect of KD025 on adipocyte differentiation is through the inhibition of CK2. Both CX-4945 and KD025 suppressed the generation of lipid droplets and the expression of proadipogenic genes *Pparg* and *Cebpa* in 3T3-L1 cells during adipocyte differentiation. Fasudil exerted no significant effect on the quantity of lipid droplets, but another ROCK inhibitor Y-27632 increased the expression of *Pparg* and *Cebpa*. Both CX-4945 and KD025 acted specifically in the middle stage (days 1–3) but were ineffective when treated at days 0–1 or the late stages, indicating that CX-4945 and KD025 may regulate the same target, CK2. The mRNA and protein levels of CK2α and CK2β generally decreased in 3T3-L1 cells at day 2 but recovered thereafter. Other well-known CK2 inhibitors DMAT and quinalizarin inhibited effectively the differentiation of 3T3-L1 cells. Taken together, the results of this study confirmed that KD025 inhibits ROCK2 and CK2, and that the inhibitory effect on adipocyte differentiation is through the inhibition of CK2.

## 1. Introduction

Rho-associated coiled-coil-containing protein kinases (ROCKs/Rho-kinases) are serine/threonine kinases introduced as downstream binding partners of RhoGTP protein [1,2]. To date, two major isoforms of ROCK, namely, ROCK1 (ROKβ) and ROCK2 (ROKα), have been studied intensively. They have a similarity of 65% of overall amino acid identity and 92% in the kinase domain [3]. ROCKs contribute to various cellular functions by regulating actin cytoskeleton, including cell contraction and movement, and adhesion [3]. They also regulate energy balance and glucose metabolism [4,5], cell proliferation [6,7], and apoptosis [8,9].

In cell culture model system, Rho/ROCK activity negatively regulates adipocyte differentiation. Unspread and round cell shape [10,11] and insulin-stimulated activation of Akt [12,13,14] are a prerequisite for commitment to adipocyte fate. In this context, the constitutive activation of the RhoA-ROCK pathway induces osteogenesis, whereas the inhibition of ROCKs using Y-27632 or fasudil promotes adipogenesis in various cell types, such as 3T3-L1, mouse embryonic fibroblast, mesenchymal stem cells (MSCs), human adipose-derived stem cells (hADSCs), periodontal ligament stem cells, and osteosarcoma-initiating cells [10,15,16,17,18,19,20,21]. Previously, we also confirmed that potent ROCK inhibitors H-1152P and SR3677 play an adipogenic role in hADSCs [19]. In specific, ROCK2 isoform has been suggested to be responsible for this adipocyte fate determination [15]. These studies show that ROCK inactivation provides an environment suitable for the differentiation of precursor cells into adipocytes by reorganizing the cytoskeletal structure and by increasing insulin-induced Akt activity.

ROCKs are involved in numerous diseases, including neuronal injury, cardiovascular diseases, pulmonary hypertension, ocular disease, and metabolic diseases [22]. Hence, several studies have focused on the development of ROCK-targeting agents for decades. Currently, fasudil and ripasudil are being prescribed as therapeutics in Japan and China [23]. However, ROCK1 and ROCK2 serve redundant functions in actin cytoskeletal organization and distinct isoform-specific functions. Thus, isoform-specific inhibitors that can reduce side effects caused by the simultaneous inhibition of both inhibitors must be urgently developed. In this respect, KD025 (SLx-2119, belumosudil) has been attracting attention as a ROCK2-specific inhibitor with IC_50_ = 105 nM (24 µM for ROCK1) [24]. KD025 has various therapeutic potentials in the treatment of cerebral ischemia [25] and autoimmune disease [26] and is in ongoing or completed phase II clinical trials for chronic graft-versus-host-disease (cGvHD), idiopathic pulmonary fibrosis, and psoriasis [27]. Moreover, we observed that the oral bioavailability of KD025 is moderate with absolute bioavailability of 37% in a rat model [28].

In previous studies, we discovered that KD025 suppresses the adipocyte differentiation of 3T3-L1 and hADSCs by downregulating key adipogenic/lipogenic genes, such as PPARγ and C/EBPα [19,20]. This inhibitory effect of KD025 on adipocyte differentiation is the opposite that of other ROCK inhibitors. The antiadipogenic effect of KD025 is specific to differentiation stage as evidenced by the minimal effect of KD025 treatment at an late stage. Importantly, the antiadipogenic regulation by KD025 outperforms the proadipogenic effect derived from ROCK inhibition. Most kinase inhibitors and KD025 bind competitively to well-conserved ATP binding sites and thus may represent polypharmacology that causes off-target effects [29]. Therefore, in this study, we inferred that KD025 regulates adipocyte differentiation by inhibiting a key regulator(s) other than ROCK2 and screened novel targets of KD025 by using the KINOMEscan^TM^ screening platform. Here, we introduce casein kinase 2 (CK2) as a novel target of KD025 and provide evidence that CK2 inhibition mediates the antiadipogenic effect of KD025 in 3T3-L1 cells.

## 2. Results

### 2.1. Identification of Novel Targets of KD025

**Figure 1:**
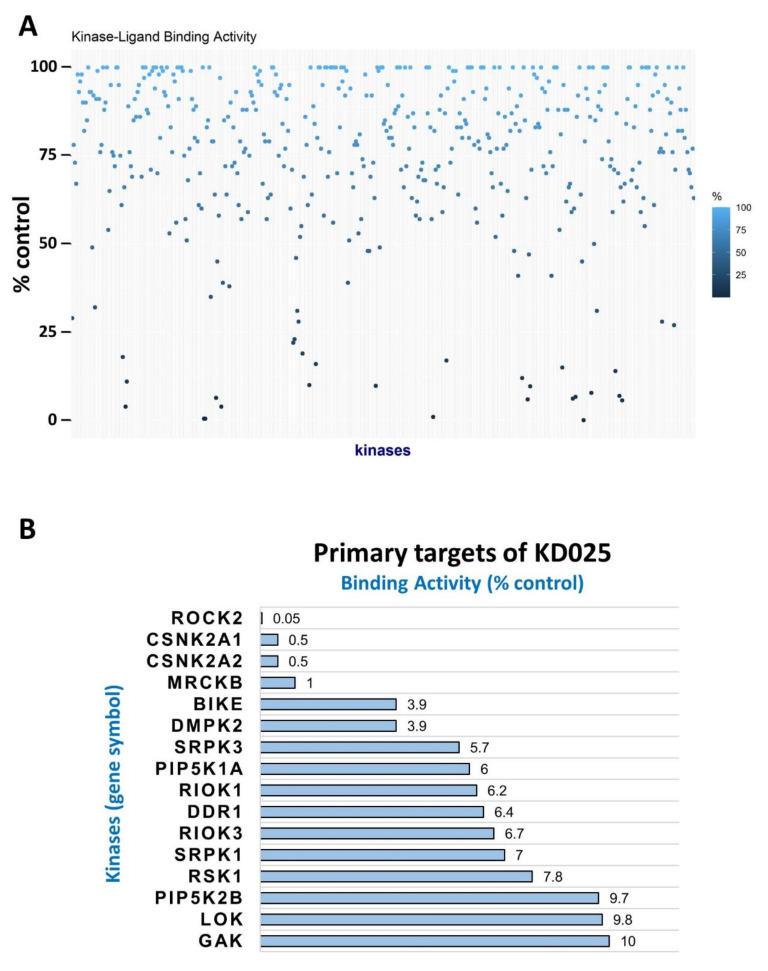

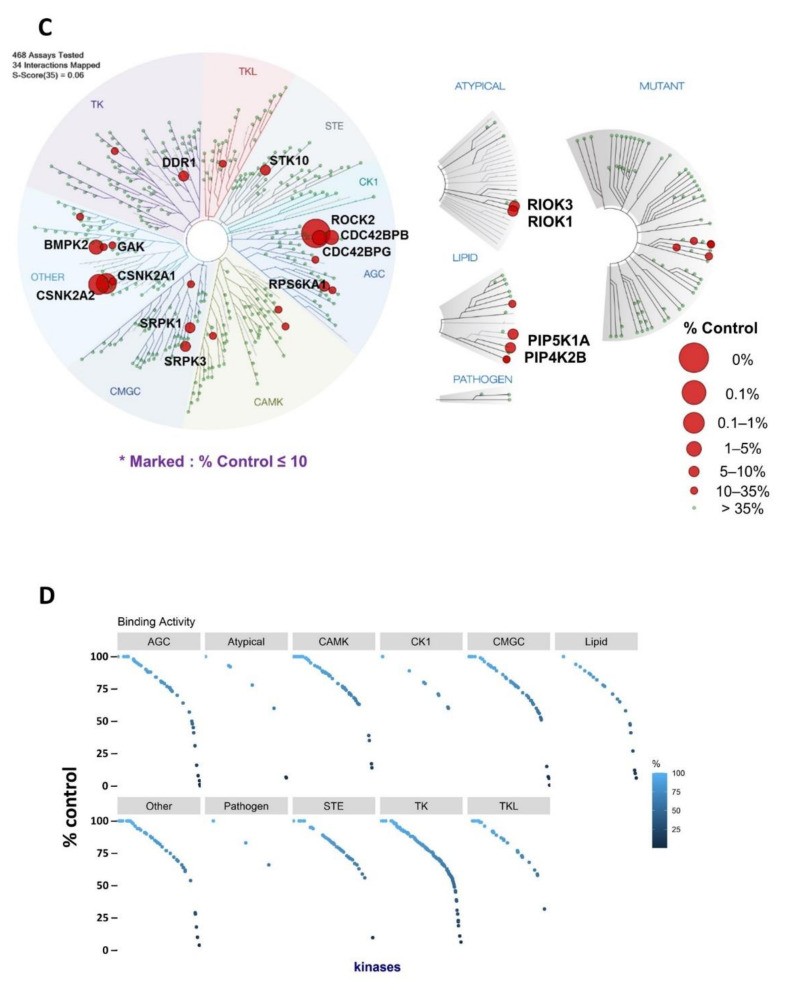

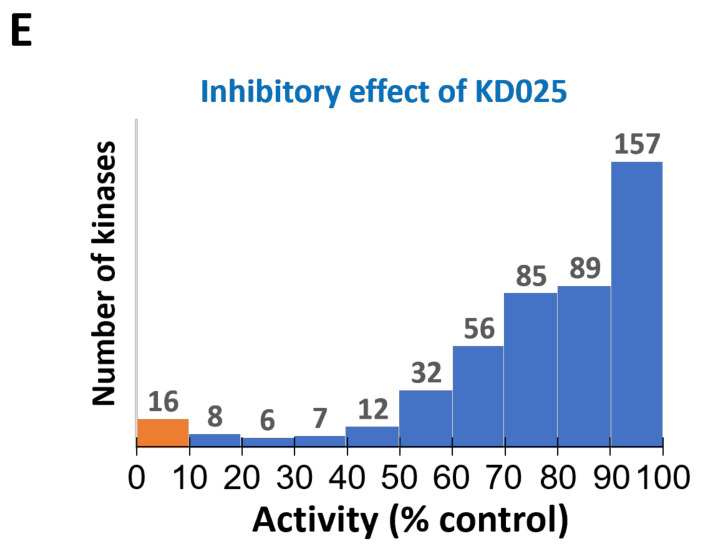
Kinome-wide target identification of KD025. Targets of KD025 (10 μM) were screened against 468 recombinant human kinases (more than 80% coverage) by using the KINOMEscan^TM^ screening platform. The experiment was performed in duplicate. (**A**) KD025 reduced kinase-ligand binding and the remained binding activity of kinases were plotted. Darker dots located near at the bottom indicate strong inhibition of KD025. Kinases were arranged in alphabetical order on the x-axis. (**B**) Top-ranked target kinases ≤ 10% ligand binding activity. ROCK2, the original target of KD025, showed the lowest activity remained (0.05%). (**C**) KD025–kinase interaction maps for 468 human kinases. Kinases binding with KD025 were marked by red circles; the larger the circles, the higher binding affinity with KD025. The kinase dendrogram was provided from KINOMEscan^TM^ service. The name of kinases with high affinity (POC ≤ 10%) was marked near the corresponding circles. (**D**) Familial distributional pattern of kinase-ligand binding activity affected by KD025. Kinases were sub-grouped by kinase family and sorted by POC. AGC, atypical; CAMK, calcium/calmodulin-dependent kinases; CK1, casein kinase 1 family; CMGC; lipid; other; pathogen; STE; TK, nonreceptor tyrosine kinases; TKL, tyrosine kinase-like kinases. (**E**) Histogram of KD025-kinase binding. X-axis indicates remained kinase-ligand binding activity, and y-axis shows the number of kinases belonging to each bin. Bin size = 10%.

We screened a panel of 468 recombinant human protein kinases to discover novel targets of KD025 by using the competition binding assay approach. The inhibitory effect of KD025 was indicated as percent of control (POC) (Figure 1A). The whole POC profiles of KD025 on tested kinases are provided in Supplementary Table S1. Around 3% of kinases (16 out of 468) showed strong binding (POC ≤ 10%) with KD025 (Figure 1B). As expected, ROCK2, a known primary target, was the most severely disturbed in substrate binding (POC = 0.05), proving the upmost specificity of KD025. By contrast, the binding activity of ROCK1 was still remained by 45% (900-fold vs. ROCK2 in POC), showing strong selectivity of KD025 over the ROCK2 isotype. CK2α and CK2α’ subunits (gene name: *CSNK2A1* and *CSNK2A2*) were the next highly interacting proteins with KD025 (POC = 0.5 in both), even though they were inhibited 10-fold less than ROCK2.

The dendrogram and clustering along with the major kinase groups (AGC, CAMK, CK1, CMGC, lipid, other, pathogen, STE, TK, TKL, and atypical groups) showed no clear preference for a specific group (Figure 1C,D). KD025 showed strong inhibitory power against only a group of kinases while showing weak inhibitory power against most of the others (Figure 1E). The selectivity scores (S-scores), of which lower level indicates higher selectivity of a compound, were S(35) = 0.069, S(10) = 0.037, and S(1) = 0.007. In short, KD025 is a highly selective inhibitor having inhibitory activities against ROCK2 and CK2α/α’.

### 2.2. Binding and Inhibitory Activities of KD025 on CK2α

**Figure 2:**
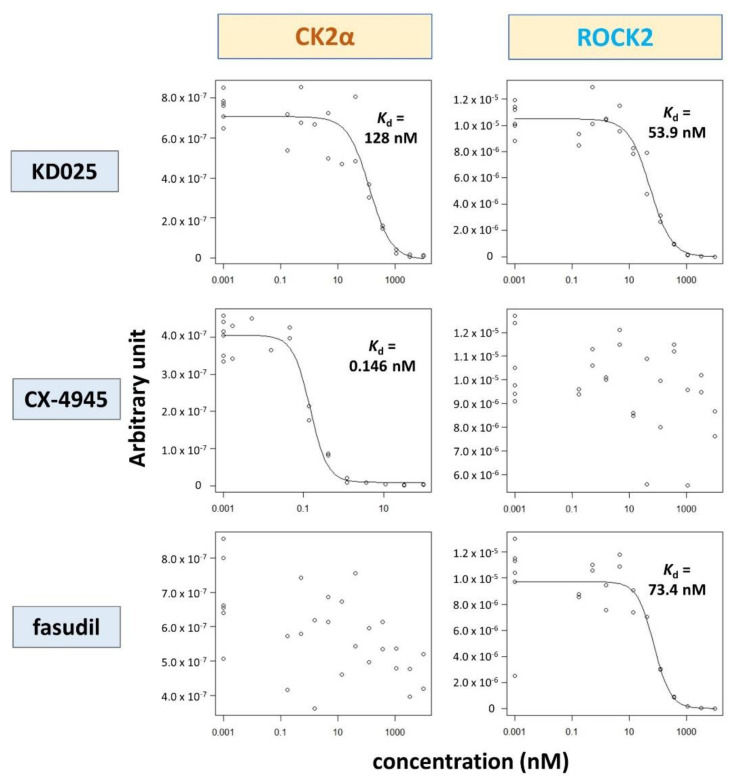
Determination of dissociation constant (*K*_d_). Interactions between drugs (KD025, CX-4945, and fasudil) and kinases (CK2 and ROCK2) were measured by applying the KINOMEscan^TM^ profiling platform. The remained kinase-ligand binding activities were measured for 11-point concentrations of drugs. *K*_d_ was calculated based on the nonlinear least-square fit model using the “drm” function of drc package in R software. Outliers were excluded in the model construction. The experiment was performed in duplicate.

We measured the *K*_d_ values of KD025 for ROCK2 and CK2α to be approximately 54 and 128 nM, respectively (Figure 2). By contrast, CX-4945, a known CK2α-specific inhibitor, had a *K*_d_ value of about 0.15 nM for CK2α but had no selective interaction with ROCK2. Meanwhile, ROCK inhibitor fasudil had a *K*_d_ value of 73 nM for ROCK2 but no interaction with CK2α. These results demonstrate that, different from other ROCK or CK2 inhibitors, KD025 could target ROCK2 and CK2α efficiently.

**Figure 3:**
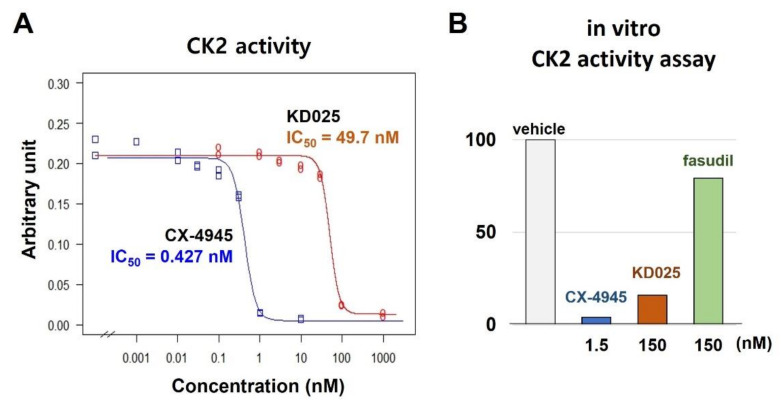
Measurement of in vitro inhibitory efficacy of KD025 against CK2. CK2 activity assay based on the ELISA system was performed using KD025 as an inhibitor. (**A**) Enzymatic activity was measured at the eight-point concentration (0, 0.1, 1, 3, 10, 30, 100, and 1000 nM for KD025; 0, 0.001, 0.01, 0.03, 0.1, 0.3, 1, and 10 nM for CX-4945) of drugs. IC_50_ was calculated by constructing a dose-response curve using the nonlinear least-square fit model. CX-4945 was used as a positive control. The experiment was performed in duplicate. (**B**) Inhibitory effects of KD025 were compared with pan-ROCK inhibitor fasudil. Higher concentration (150 nM) than experimentally determined IC_50_ of KD025 was applied for KD025 and fasudil. Potent CX-4945 was tested at 1.5 nM. The assay was performed in singlet.

We performed a CK2 activity assay to confirm the inhibitory efficacy of KD025 against CK2α. Results showed that KD025 inhibited CK2 activity efficiently in a nanomolar range (IC_50_ = 49.7 nM), although CX-4945 had much potent inhibitory activity (IC_50_ = 0.427 nM) than KD025 (Figure 3A). By contrast, fasudil inhibited minimally CK2 effectively even at a relatively high concentration (150 nM) (Figure 3B).

### 2.3. CK2α Shows Low Sequential and Structural Similarities with ROCK2

**Figure 4:**
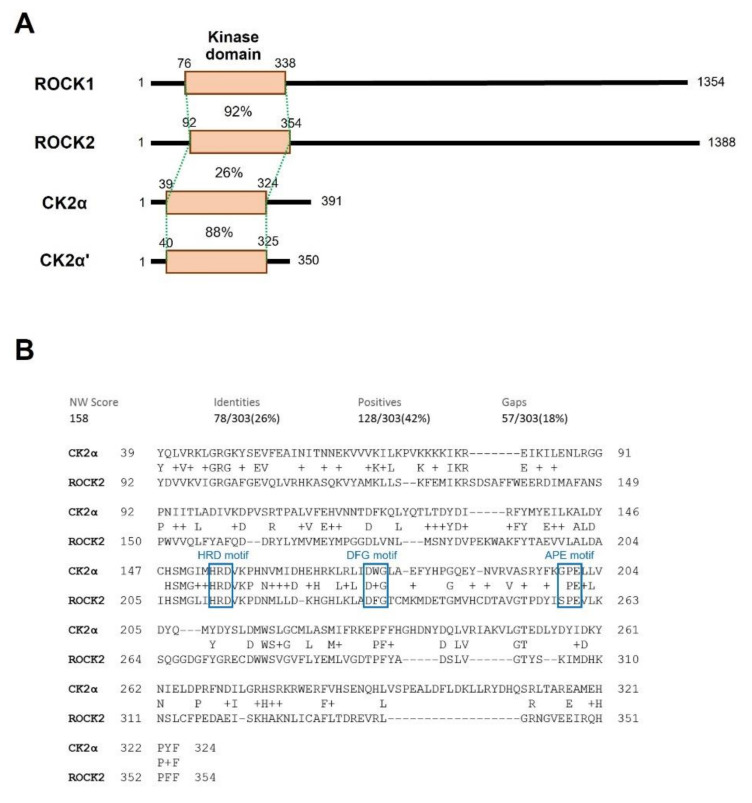

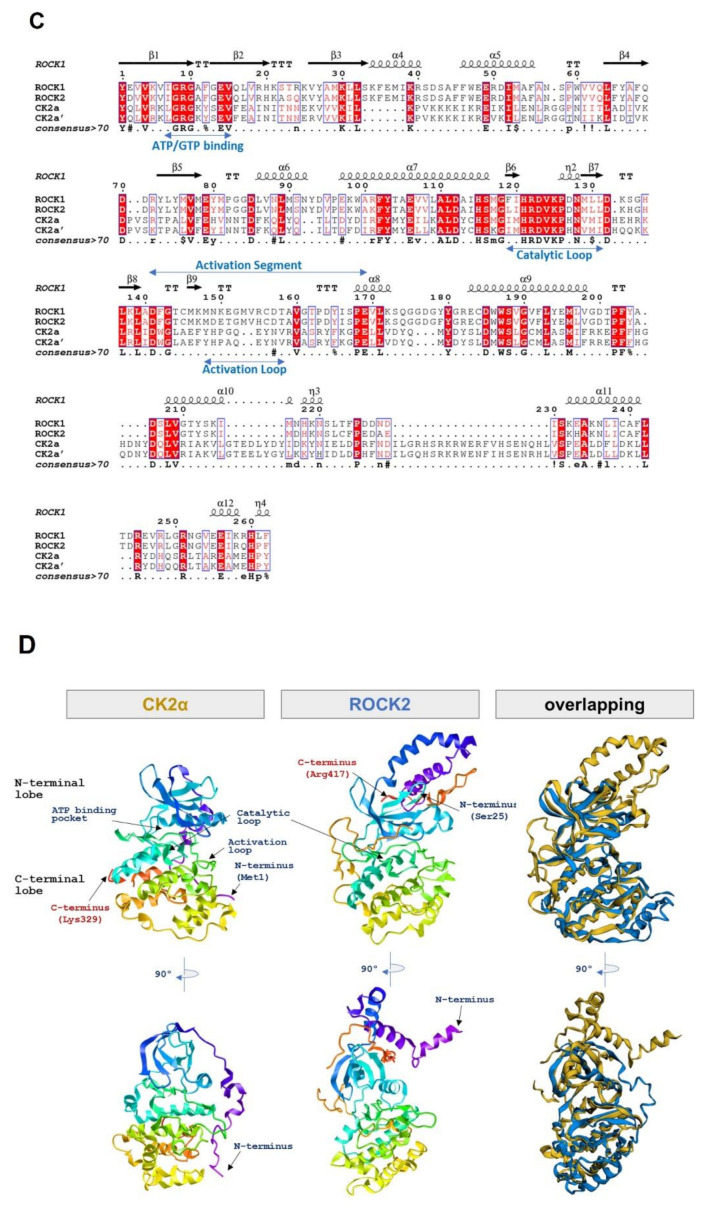
Sequence alignment and structural comparison of CK2 and ROCK2. (**A**) Structure of ROCKs and CK2 isotypes. ROCKs are relatively long but CK2 isotypes are short. ROCKs have the kinase domain at N-terminal region which is a conserved region among kinases. (**B**) Sequence alignment of the kinase domain of CK2α (aa 39–324) and ROCK2 (aa92–354) by the Needleman–Wunsch global alignment algorithm using BLAST software. Several known kinase motifs were indicated in blue color box (the HRD, DFG, and APE motifs). (**C**) Multiple sequence alignment of human ROCKs and CK2 proteins in the kinase domain. The alignment was performed using COBALT alignment tool and the result was displayed using ESPript (Version 3.0, http://espript.ibcp.fr/, accessed on 4 August 2021). The kinase domains were used for multiple alignment as described in Table 1. The Secondary structural elements for ROCK2 are displayed above and the consensus sequences are noted below sequences. Several conserved motifs were marked by bidirectional arrows in blue color with descriptions. (**D**) Structural alignment of CK2α with ROCK2 covering the kinase domain. 3D structures of CK2α (left, PBD ID: 6YPN, aa 1–329) and ROCK2 (middle, PBD ID: 7JOV, aa 23–417) were displayed using iCn3D structure viewer (https://www.ncbi.nlm.nih.gov/Structure/icn3d/full.html, accessed on 4 August 2021). The structural similarity and the overlapping structure (right) were calculated by FATCAT software (https://fatcat.godziklab.org/, accessed on 4 August 2021).

ROCK2 belongs to the group ‘AGC’, whereas CK2α belongs to ‘OTHER’ kinases. Human CK2α and CK2α’ contain the core kinase domain and short additional sequences on both ends. Thus, their overall size is relatively small (391 and 395 aa respectively) and they are constitutively active. On the other hand, ROCK1 and ROCK2 are large (1354 and 1388 aa) as they contain the large structural and regulatory domains at the C-terminus. The structures of ROCKs and CK2 subunits are compared to each other with a description of the kinase domain (Figure 4A).

Since KD025 interacts and inhibits both ROCK2 and CK2α, we examined whether these kinases have a sequence similarity to one another. The general information on the kinases used for comparison is presented in Table 1. The sequence similarity between ROCK1 and ROCK2 was 64% overall (not shown) and 92% in the kinase domain (Figure 4A and supplementary Figure S1). The similarity between CK2α and CK2α’ was 88% in this domain. In contrast, the sequence similarity between ROCK2 and CK2α in the kinase domain was much lower (26%) (Figure 4A,B). Multiple sequence alignment of ROCK1, ROCK2, CK2α, and CK2α’ revealed that the sequences are less conserved between ROCKs and CK2s (Figure 4C).

Nevertheless, sequences known to be critical for a phosphorylation reaction, such as ATP/GTP binding loop and the catalytic loop (HRD motif) are highly conserved among these kinases. In contrast, the sequences of the activation loop (DFG-APE motif) that controls kinase activity are highly variable between ROCKs and CK2s. Their structural difference was also evident in the three-dimensional (3D) alignment of CK2α and ROCK2. The 3D structures of ROCK1 and ROCK2 overlap each other well (RMSD = 0.94 Å, Figure S2, in the Supplementary Materials), but CK2α had a lot of areas that did not match ROCK2 (RMSD = 2.56 Å, Figure 4D). These data collectively show that CK2 and ROCK2 have relatively low structural similarity throughout their entirety, except for the sequences essentially required for kinase activity. Considering that KD025 does not effectively inhibit ROCK1, which has high sequence and structural similarity with ROCK2 in kinase domains, the possibility that the KD025′s binding site to CK2α is not relevant to that to ROCK2, cannot be excluded. Investigation of the crystallographic structure of KD025-bound CK2α and ROCK2 may be necessary to understand the exact mechanism.

### 2.4. Downregulation of Adipocyte Differentiation ss Mediated by CK2 Inhibition

**Figure 5:**
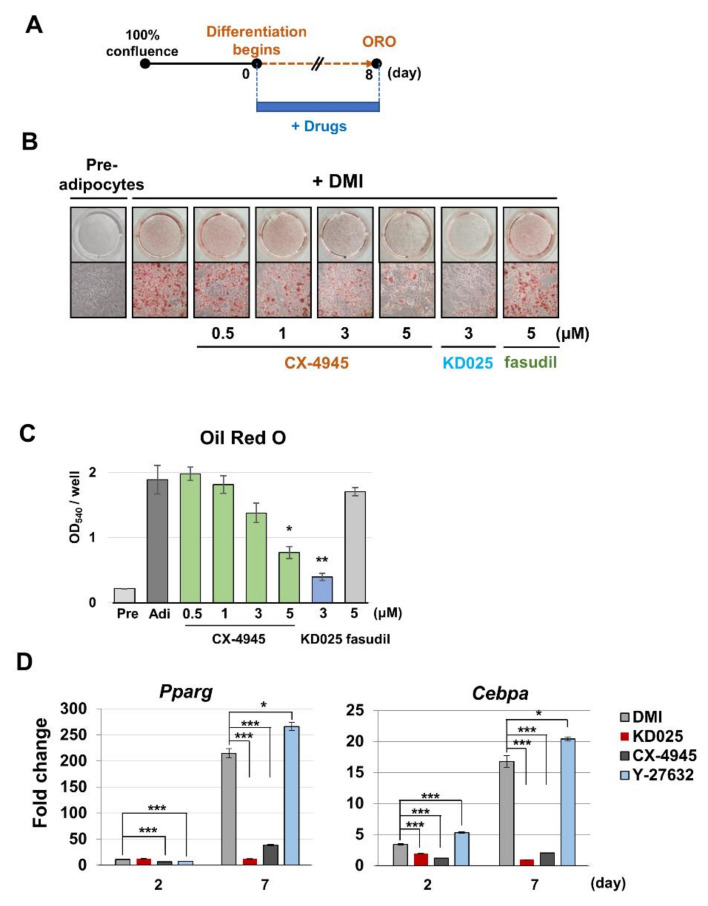
Effect of CX-4945 on adipogenesis in 3T3-L1 cells. 3T3-L1 cells were differentiated by incubating in differentiation media (DMI) with or without CX-4945 at various concentrations. KD025 (3 μM) and fasudil (5 μM) were used as positive and negative controls. (**A**) Experimental scheme of differentiation and sample preparation. (**B**) At day 8, cells were stained using ORO and microscopic images were taken for ORO-stained cells. (**C**) Lipid accumulation was assessed by obtaining absorbance at 540 nm. (**D**) KD025 (5 μM), CX-4945 (10 μM) and Y-27632 (10 μM) were treated. Total RNA was extracted from the differentiating cells at days 0, 2, and 7. qRT-PCR analysis was performed for adipogenic genes *Pparg* and *Cebpa*. The level was calculated by the ΔCt method and expressed as fold changes compared with the value of preadipocyte (day 0). * *p* < 0.05; ** *p* < 0.01; *** *p* < 0.005 vs. DMI control at the same day.

We have previously shown that KD025 inhibits the adipocyte differentiation of 3T3-L1 cells and human-originated cultured ADSCs [19,20]. In those studies, ROCK2 inhibition is not associated with the antiadipogenic effect of KD025, indicating that an unknown KD025 target other than ROCK2 might control adipogenesis. Interestingly, several studies have suggested CK2 as a proadipogenic regulator [30,31,32,33,34]. Therefore, we deduced that CK2, not ROCK2, is an important proadipogenic regulator that could be targeted by KD025 in cultured cell models. To test this hypothesis, we treated various concentrations of CX-4945 in differentiating 3T3-L1 cells (from day 0 to day 7) (Figure 5A). Oil Red O (ORO) staining showed that the administration of CX-4945 significantly reduced the amount of accumulated fat in a dose-dependent manner (Figure 5B,C). As previously reported, KD025 reduces fat accumulation, whereas fasudil does not [19,20]. These results imply that CK2 activity is required for the differentiation of 3T3-L1 preadipocytes and that KD025 may target CK2, not ROCK2, to exhibit antiadipogenic activity. Then, we tested if the inhibition of CK2 is responsible for suppressing the expression of *Pparg* and *Cebpa* genes [19,20]. Similar to KD025, treatment with CX-4945 markedly suppressed the expression of *Pparg* and *Cebpa* genes during differentiation (Figure 5D). Y-27632, a pan-inhibitor for ROCKs, did not suppress the expression of *Pparg* and *Cebpa* genes, indicating that the inhibition of ROCKs was not related to the anti-adipogenic effect of KD025.

### 2.5. KD025 and CX-4945 Regulate the Intermediate Stage of Adipogenesis

**Figure 6:**
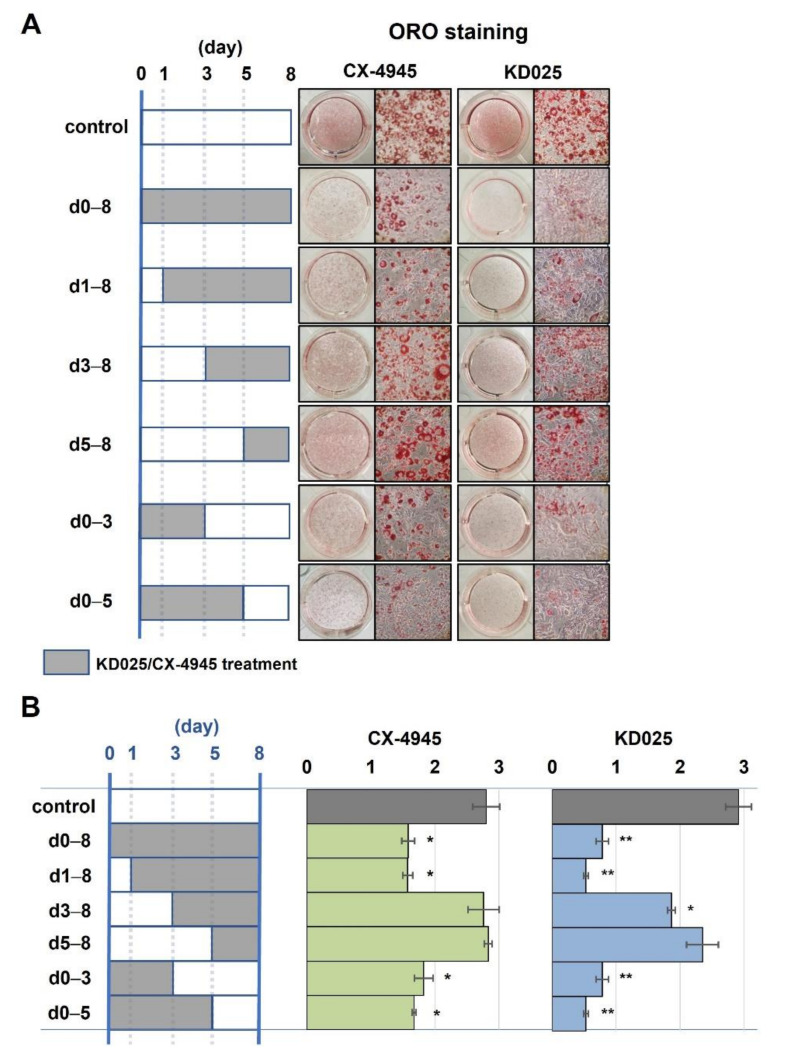
Parallel comparison of stage-specific effect between CX-4945 and KD025 on the adipogenesis of 3T3-L1 cells. 3T3-L1 cells were treated CX-4945 (5 μM) or KD025 (3 μM) at various times as indicated by grey bar during differentiation. At day 8, cells were stained with ORO. (**A**) Microscopic images were taken, and (**B**) lipid accumulation was assessed by obtaining absorbance at 540 nm. * *p* < 0.05; ** *p* < 0.01 vs. vehicle.

The periodic mode of action of KD025 was compared with that of CX-4945 to examine whether or not the antiadipogenic effect of KD025 is mediated by CK2 inhibition. As shown in Figure 6A,B, the quantity of lipid droplets significantly reduced when KD025 or CX-4945 was incubated during the intermediate stage (days 1–3). The treatment of inhibitors during the early (days 0–1) or late (days 5–8) stage had lesser effect. Importantly, CX-4945 showed a highly similar periodic pattern to KD025, indicating that KD025 regulates the same target as CX-4945.

### 2.6. CK2 Is a Key Regulator in Adipogenesis

**Figure 7:**
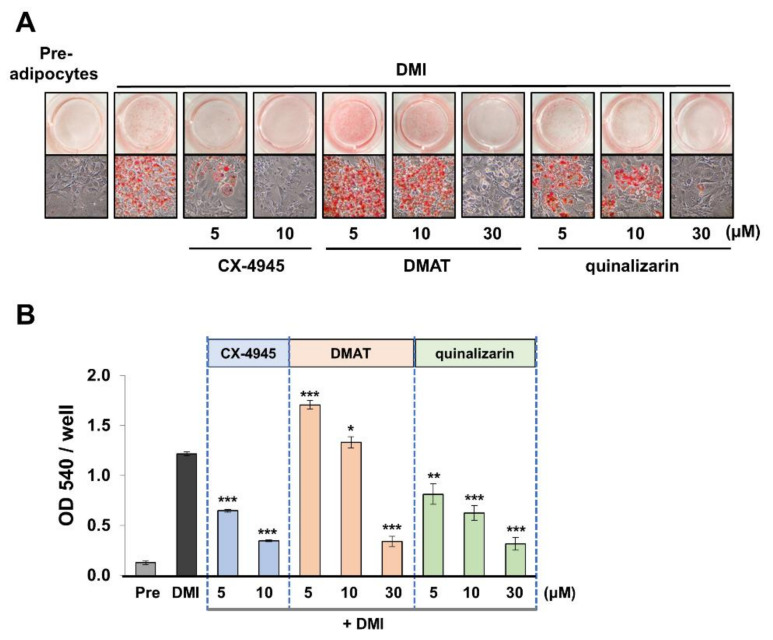
Effect of CK2 inhibitors on the adipocyte differentiation of 3T3-L1 cells. 3T3-L1 cells were treated with CX-4945, DMAT or quinalizarin during adipocyte differentiation and stained with ORO at day 8. (**A**) Microscopic images were acquired, and (**B**) lipid accumulation was assessed by obtaining absorbance at 540 nm. * *p* < 0.05; ** *p* < 0.01; *** *p* < 0.005 vs. DMI vehicle.

To further verify the role of CK2 in adipogenesis, we tested the effect of different classes of CK2 inhibitors on adipogenesis: DMAT (2-dimethylamino-4,5,6,7-tetrabromo-1H-benzimidazole) (class benzoimidazoles) and quinalizarin (class anthraquinones). Quinalizarin is a potent inhibitor for CK2 holoenzyme (58 nM) and is more selective than other anthraquinones [35]. Importantly, it inhibits CK2 efficiently at the concentration that marginally affects ROCK2 [36]. Besides, DMAT is a widely studied CK2 inhibitor having a benzoimidazole skeleton. DMAT has relatively higher promiscuity score among CK2 inhibitors [37]: Quinalizarin has 9.47% of promiscuity score [36], whereas DMAT has 48.60% [38]. Using these inhibitors, we tested if the adipocyte differentiation is modulated by controlling CK2 activity. ORO staining results showed that quinalizarin suppressed adipogenesis at a wide range of concentrations (5, 10, and 30 μM) (Figure 7A,B). However, the less selective inhibitor DMAT did not show consistent inhibitory activity against adipogenesis: it inhibited the adipogenesis of 3T3-L1 only at the highest concentration (30 μM) while promoting reversely the differentiation at the lowest concentration (5 μM). These results indicate that DMAT inhibits various kinases depending on the concentration used. Thus, the outcomes result from combined effects. In short, our study showed that KD025 is a dual inhibitor for ROCK2 and CK2 with high potency and that CK2 is the main mediator associated with the control of 3T3-L1 cell differentiation.

**Figure 8:**
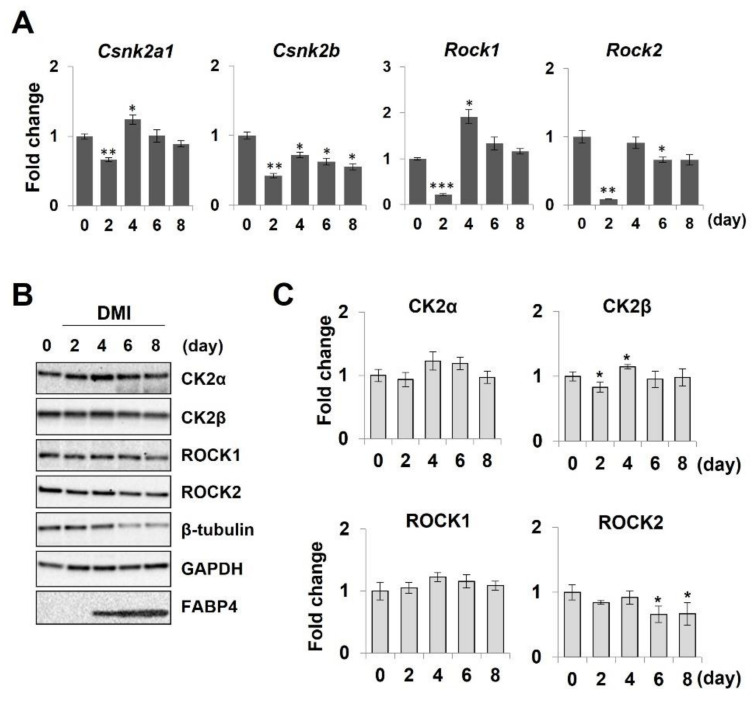
mRNA and protein expression levels of ROCKs and CK2 during the adipogenesis of 3T3-L1 cells. Differentiating 3T3-L1 cells were harvested on various time points (days 0, 2, 4, 6, and 8). (**A**) mRNA expression levels of *Rock1*, *Rock2*, *Cskn2a1*, and *Cskn2b* were assessed by qRT-PCR. * *p* < 0.05; ** *p* < 0.01; *** *p* < 0.005 vs. day 0. (**B**) Protein expression levels of ROCK1, ROCK2, CK2α, and CK2β were visualized by immunoblot. FABP-4 was used as a differentiation marker. β-tubulin and GAPDH were used for loading controls. (**C**) The band intensity of CK2 subunits and ROCKs were quantified. * *p* < 0.05; ** *p* < 0.01; *** *p* < 0.005 vs. day 0.

During the adipogenesis period, the mRNA levels of the *Csnk2a1* (CK2α) and *Csnk2b* (CK2β), *Rock1* and *Rock2* genes were all downregulated on day 2, recovered on day 4, and then maintained with small variability (Figure 8A). Compared with mRNA levels, their protein levels were more constant with small changes (Figure 8B,C). In particular, the mRNA level significantly reduced on day 2, whereas the amount of protein showed only minimal variability.

## 3. Discussion

ROCK2-specific inhibitor KD025 has a comparable degree of binding and inhibitory ability against CK2 and ROCK2. Our results suggest that KD025 may produce combinatorial effects resulting from the dual inhibition of ROCK2 and CK2. Although the interaction between KD025 and ROCK2 was stronger, the interaction between KD025 and CK2 was strong enough, as detected at the nanomolar concentration level. In addition, the inhibitory effect of CK2 was clearly revealed at the cellular level. Furthermore, we confirmed for the first time the mechanism by which KD025 inhibits adipocyte differentiation while inhibiting ROCK2. ROCKs inhibit adipocyte differentiation, whereas CK2 promotes adipocyte differentiation. Treatment with ROCK inhibitors decreases ROCK activity and thus promotes adipocyte differentiation. Conversely, treatment with CK2 inhibitors downregulates adipocyte differentiation due to decreased CK2 activity. KD025 inhibits adipocyte differentiation by simultaneously inhibiting ROCK2 and CK2. These findings indicate that, although the inhibitory function of ROCK2 is reduced, the intact activity of CK2 is still required to proceed with adipocyte differentiation.

The fact that KD025 can inhibit targets other than ROCK2 is important in various aspects in ROCK-targeted therapeutics. ROCKs are targets in the treatment of various diseases. Historically, ROCKs are downstream signaling molecules of RhoGTPase and introduced to mediate the formation of actin cytoskeleton [1,2]. Although they function redundantly in the regulation of cytoskeleton and cell cycle progression/tumorigenesis [7], ROCK1 and ROCK2 isoforms differ in structures, have distinct binding partners, and play isoform-specific roles [3]. For instance, ROCK2 isoform promotes the proinflammatory environment. ROCK2 regulates T-cell plasticity and macrophage polarization by enhancing the production of proinflammatory cytokines, such as IL-21 and IL-17, in CD4^+^ T-cells of mouse model [39]. Targeted inhibition of ROCK2 or KD025 treatment ameliorates the clinical symptoms of chronic cGvHD in murine models [26,40]. In addition, ROCK2 plays a key role in macrophage polarization into proinflammatory macrophage type 1 in age-related macular degeneration model [41].

Recent studies have indicated that ROCK1 and ROCK2 isoforms play important roles in metabolism. In mice, global ROCK1-deletion triggers whole body insulin resistance by impairing skeletal muscle insulin signaling [42]. In addition, ROCK1 activity is significantly reduced in the skeletal muscle of human subjects with obesity and type 2 diabetes [43]. In accordance, ROCK1 mediates insulin-stimulated glucose transport in adipose and muscle cells [5]. However, the role of ROCK1 remains controversial in the literature. Global and adipose-specific ROCK1-deleted mice show enhanced insulin response in adipose tissue and improved insulin sensitivity in obese mice, respectively [44]. Other studies showed that some ROCK inhibitors, such as Y-27632 and fasudil, enhance the insulin-Akt signaling pathway in various cell types [15,19,45]. These reports indicate that the roles of ROCK1 in glucose metabolism and insulin signaling depending on the context of examined objects.

By contrast, the role of ROCK2 in glucose metabolism is less studied than that of ROCK1. ROCK2 functions differently from ROCK1 in glucose metabolism in that ROCK2 directly interacts and phosphorylates IRS-1 in cells [5]. ROCK2-partially deleted (ROCK2^+/−^) mice show a lean body mass phenotype and improved insulin sensitivity during aging [46]. ROCK2 suppresses adipocyte differentiation in 3T3-L1 and hADSCs, whereas ROCK1 performs no special function [15,19]. Several pan-ROCK inhibitors, including Y-27632, fasudil, H-1152P, and SR3677, enhance adipocyte differentiation in those models.

We have previously shown that KD025 inhibits the differentiation of 3T3-L1 cells and hADSCs into adipocytes [19,20]. Bioinfomatics analysis also demonstrated that KD025 plays a regulatory role in adipogenesis [47]. However, the inhibition of adipocyte differentiation by KD025 has the opposite outcome resulting from the inhibition of ROCK2 activity, suggesting that KD025 controls other important differentiation regulatory factors aside from ROCK2. In this study, we revealed that CK2 is a novel target of KD025. CK2 is a serine/threonine-selective protein kinase that participates in various biological processes, such as cell cycle progression, transcription/translation, protein stability, and regulation of the circadian rhythm (reviewed in [48]). Dysfunction of CK2 has been linked to tumorigenesis in diverse cancers. Currently, CK2 inhibitor CX-4945 is under clinical trials for cancer treatment [27]. CK2 is typically a tetrameric complex composed of two catalytic subunits (α and α’) and two identical regulatory β subunits. The catalytic unit can be formed in a homodimer (α & α, or α’ & α ‘) or heterodimer (α & α’).

Diverse pieces of evidence indicate that CK2 serves proadipogenic and antithermogenic functions. CK2 activity is required for insulin-Akt signal transduction; its inhibition by CX-4945 suppresses insulin-mediated glucose uptake and Akt activation in 3T3-L1 adipocytes and human primary adipocytes [30]. CK2 protein level and activity increase in white adipose tissue in ob/ob and db/db mice and obese humans [30]. CK2 activity is preferentially higher in white adipocytes than in brown/beige adipocytes, and the genetic or pharmacological inhibition of CK2 in white adipocytes induces beige adipocyte biogenesis and ameliorates diet-induced obesity in mice [49]. SIRT1 is phosphorylated by CK2 at Ser-164 and loses its deacetylase activity in obese mice, which is linked to the development of nonalcoholic fatty liver disease [50].

CK2 activity is also required for the progress of adipocyte differentiation in 3T3-L1 [33,34], mouse C3H/10T1/2 cells [32], and human MSCs [31]. CK2 protein level and activity are maintained in 3T3-L1 cells during the early-to-mid adipogenic stage [34,36] but are reduced during most of the differentiation period in hMSCs [31]. CK2 inhibitors, such as quinalizarin, CX-4945, and DMAT, suppress adipocyte differentiation in 3T3-L1 and hMSCs [9,31,34], and we also confirmed the antiadipogenic effect in this study.

However, the mechanism by which CK2 regulates the differentiation of adipocytes remains unclear. CK2 possibly phosphorylates several HDACs, including HDAC1, HDAC2, and SIRT1 [49,50,51,52], which may be involved in adipocyte differentiation through various mechanisms that have not been well documented until now. This subject is expected to be revealed by various studies in the future.

## 4. Materials and Methods

### 4.1. Inhibitors

KD025 [2-(3-(4-((1H-indazol-5-yl)amino)quinazolin-2-yl)phenoxy)-N-isopropylacetamide] and fasudil were purchased from Medchem Express (NJ, USA), Y-27632 from Selleck Chemicals (TX, USA). CX-4945 and DMAT were provided by Medchem Express (NJ, USA). Quinalizarin (1,2,5,8-tetrahydroxyanthraquinone) was purchased from Sigma-Aldrich (Sigma-Aldrich, MA, USA).

### 4.2. Drug-Kinase Binding Assay

To screen targets of KD025, we applied the KINOMEscan^TM^ screening platform (Eurofins DiscoverX, CA, USA), which is an active site-directed competition binding assay to measure quantitatively the interactions between test compounds and kinases. This platform contains 468 recombinant human kinases (more than 80% coverage) [53] in which 56 disease-relevant mutants are included. The recombinant DNA-tagged kinases were incubated with the probe ligands immobilized to a solid support, and then the level of the captured kinases on the solid support was measured using quantitative PCR. The binding ability of KD025 (10 μM) with a kinase was accessed by measuring the amount of kinases remained in the solid support and calculating the POC by the Equation below, where lower numbers indicate stronger hits:

(1) $$\mathrm{POC}=\left[\frac{\mathrm{signal}\left(\mathrm{KD}025-\mathrm{treated}\right)-\;\mathrm{signal}\left(\mathrm{positive}\;\mathrm{control}\right)}{\mathrm{signal}\left(\mathrm{DMSO}\right)-\;\mathrm{signal}\left(\mathrm{positive}\;\mathrm{control}\right)}\right]\times 100$$

To describe the selectivity of KD025 for a kinase, we calculated the selective score (S-score) as follows:

S (x) = [number of nonmutant kinases with POC < x]/[total number of nonmutant kinases tested (403)](2)

### 4.3. Dissociation Constant Measurement

Dissociation constant (*K*_d_) was measured using KINOMEscan^TM^ profiling service. In brief, 11-point concentrations of threefold serial dilution of 10 μM KD025, CX-4945, and fasudil were incubated with kinases and active site directed ligand. The binding activity of inhibitors was monitored by measuring remained kinase-ligand binding activity. *K*_d_ was calculated based on the nonlinear least-square fit model by using the “drm” function of drc package [54] in R software (version 3.6.1).

### 4.4. Sequence Alignment

The kinase sequences were aligned using the BLAST Global Alignment tool for pairwise alignment and COBALT software [55] for multiple sequence alignment (https://blast.ncbi.nlm.nih.gov/, accessed on 4 August 2021). Only the kinase domains were aligned using amino acids provided in Table 1. The multiple sequence alignment result was displayed using ESPript (Version 3.0, http://espript.ibcp.fr/, accessed on 4 August 2021) [56].

### 4.5. Structural Alignment

The structural similarity was validated by pairwise structural alignment using FATCAT software (https://fatcat.godziklab.org/, accessed on 4 August 2021) [57].

### 4.6. CK2 Activity Assay

CK2 activity was measured using the CycLex CK2 kinase assay/inhibitor screening kit (cat# CY-1170, Medical & Biological Laboratories, Japan) in accordance with the manufacturer’s instruction. The ELISA system uses the plate coated with recombinant p53, which is phosphorylated by CK2. Peroxidase-coupled anti-phospho-p53 Ser46 monoclonal antibody detects the phosphorylation by CK2. In brief, CK2 (cat# P6010, BioLabs, MA, New England) with or without inhibitors was incubated in wells for 60 min at 30 °C, and then a detector antibody was added and incubated for 30 min at room temperature. The substrate tetra-methylbenzidine was added and incubated for 10 min at room temperature. The enzymatic activity was obtained by measuring the absorbance at 450 nm with a Synergy H1 hybrid reader (BioTek, Winooski, VT, USA). The half maximal inhibitory concentration (IC_50_) in vitro was calculated by constructing a dose-response curve using the nonlinear least-square fit model using the “drm” function of drc package in R software.

### 4.7. Cell Culture

The preadipocyte 3T3-L1 cell line was kindly provided by Dr. Hyun-Sung Park (University of Seoul) and cultured in Dulbecco’s modified Eagle’s medium (DMEM, Sigma-Aldrich) supplied with 10% heat-inactivated newborn calf serum (Invitrogen, CA, USA), 100 units/mL penicillin, and 100 µg/mL streptomycin (Cellgro, VA, USA). The cells were maintained in an incubator at 37 °C and 5% CO_2_. To initiate differentiation, the cells were incubated for additional days after 100% confluence and then further incubated in a medium containing fetal bovine serum (FBS) and a differentiation cocktail (DMI, 1 μM dexamethasone, 0.5 mM 3-isobutyl-1-methylxanthine, and 5 μg/mL insulin) as previously described [5]. In brief, at day 3, the culture medium was replaced with DMEM/10%FBS containing 5 µg/mL insulin. Then, growth medium was changed every 2 days.

### 4.8. Oil Red O Staining

Lipid droplets of cells were stained using ORO (Sigma-Aldrich, MA, USA). In brief, the cells were washed with phosphate buffered saline and incubated with 10% formalin for 5 min. The cells were then fixed with the same volume of fresh formalin for at least 1 h. Subsequently, the cells were washed with 60% isopropanol, air dried completely, and then stained with ORO working solution (1% ORO/isopropanol: distilled water = 3:2). The stained cells were washed with water four times. Cell images were taken by a microscope (Nikon Eclipse TS100-F, Japan). Then, 100% isopropanol was added and incubated for 10 min to quantify the intensity of ORO. The quantity of extracted dye was obtained by obtaining absorbance at 540 nm in the Synergy H1 hybrid reader (BioTek, VT, USA).

### 4.9. Quantitative Real-Time PCR (qRT-PCR)

Total RNA was extracted using easy-BLUE (iNtRON Biotechnology, Dajeon, Korea) in accordance with the manufacturer’s protocol. cDNA was synthesized from 2 µg of total RNA using TOPscript^TM^ Drymix Kit (Enzynomics, Dajeon, Korea). qRT-PCR was performed using TB Green ^®^ Premix Ex Taq^TM^ II (Tli RNAse H Plus) (TaKaRa, Japan) with Applied Biosystems QuantStudio™ 1 Real-Time PCR System (Thermo Fisher Scientific, Waltham, MA, USA). Gene expression data were normalized based on the use of the same amount of total RNA samples. The relative fold change of gene expression was calculated using the ∆Ct method. Sequences of primers are listed in Table 2.

### 4.10. Western Blot Analysis

Total protein was extracted from cells using lysis buffer [20 mM tris pH 7.5, 5 mM EDTA, 10 mM Na_4_P_2_O_7_, 100 mM NaF, 2 mM Na_3_VO_4_, 1% NP-40, 1 mM PMSF, and protease inhibitor cocktail (Promega, WI, USA)]. Then, 20 μg lysate was separated by SDS-PAGE. The separated proteins were transferred from the gel to a nitrocellulose membrane. The membranes were incubated with primary antibodies [ROCK1, ROCK2, CK2β, GAPDH, β-Tubulin, and FABP-4 were from Santa Cruz Biotechnology (TX, USA)]; CK2α was from Cell Signaling. The probed membranes were incubated with the secondary antibodies. The bands were detected using Amersham ECL Western Blotting Detection Reagent (GE Healthcare, IL, USA) and visualized and quantified using the ChemiDoc imaging system (Bio-Rad, CA, USA).

### 4.11. Statistical Analysis

Data for ORO staining, qRT-PCR, and immunoblot were the representatives from three independent experiments. Data are expressed as means ± S.D. based on duplicate or triplicate. * *p* < 0.05; ** *p* < 0.01; *** *p* < 0.005 vs. vehicle.

## Figures and Tables

**Table 1:** Characters of ROCKs and CK2 proteins used in structure comparison.

Kinase	NCBI ID	Length (Residues)	Molecular Weight (kDa)	Kinase Domain ^#^	Catalytic Loop	Activation Segment	PDB ID
**ROCK1**	NP_005397.1	1354	158	76–338	194–206	216–244	6E9W
**ROCK2**	NP_004841.2	1388	161	92–354	210–222	216–244	7JOV
**CK2α**	NP_001886.1	391	45	39–324	152–164	175–201	6YPN
**CK2α’**	NP_001887.1	350	41	40–325	153–165	176–202	6TGU

^#^ For comparison between ROCK and CK2 isotypes, the sequences of the CK2 kinase domain were defined after the preliminary multiple alignment using the kinase domains of ROCKs that are generally used.

**Table 2:** List of PCR primers and sequences.

Type	Gene	Primer	Primer Sequence (5′–3′)
ROCKs	*Rock1*	forward	TGCTAACCAAAGATATTGAAATGCT
		reverse	TTTATTTCTTCCTCCTTCTTCAATTT
	*Rock2*	forward	AATCTCATATGTGCCTTCTTAACAGA
		reverse	CCCAATTCCATTGATCATTCTT
CK2	*Csnk2a1*	forward	GCCGCCATATTGTCTGTGTG
		reverse	CCCTTTTTCTTCACACTGCGG
	*Csnk2b*	forward	TCCTTGGAAGCACAGCTCC
		reverse	AGTCTTCATCCACCTCACAGA
Adipogenic genes	*Pparg*	forward	TGCTGTTATGGGTGAAACTC
		reverse	CTGTGTCAACCATGGTAATT
	*Cepba*	forward	AGCTGCCTGAGAGCTCCTT
		reverse	GACCCGAAACCATCCTCTG

## Data Availability

Data is contained within the article or supplementary material.

## Supplementary Materials

Figure S1: Pairwise alignment of kinase domains between ROCK1 and ROCK2 (A) and between CK2α and CK2α’ (B). Figure S2: The structural alignment of ROCK1 and ROCK2, Table S1: KINOMEscan profiling results.

## Sample Availability

Samples of the compounds are available from the authors.
