# Supplementary material for: ROCK2-Specific Inhibitor KD025 Suppresses Adipocyte Differentiation by Inhibiting Casein Kinase 2
Source: Molecules. 2021 Aug 5;26(16):4747. doi: 10.3390/molecules26164747 (PMC8401933; doi:10.3390/molecules26164747)
Supplement: Supplementary file 1 [file molecules-26-04747-s001.zip › Supplementary figures.pdf]

SUPPLEMENTARY DATA

ROCK2-specific inhibitor KD025 suppresses adipocyte differentiation by inhibiting casein kinase 2

Nhu Nguyen Quynh Tran, Kwang-Hoon Chun*

Gachon Institute of Pharmaceutical Sciences, College of Pharmacy, Gachon University,

Incheon 21936, Republic of Korea

# Correspondence to:

Kwang-Hoon Chun, Ph.D.

Gachon Institute of Pharmaceutical Sciences, College of Pharmacy, Gachon University, Incheon 21936, Republic of Korea

Tel.: 82-32-820-4951

E-mail address: khchun@gachon.ac.kr

Supplemental Figures

(A)

NW Score	Identities	Positives	Gaps
1306	242/263 (92%)	255/263 (96%)	0/263 (0%)

```
ROCK1  76   YEVVKVIGRGAFGEVQLVRHKSTRKVYAMKLLSKFEMIKRSDSAFFWEERDIMAFANSPW  135
            Y+VVKVIGRGAFGEVQLVRHK+++KVYAMKLLSKFEMIKRSDSAFFWEERDIMAFANSPW
ROCK2  92   YDVVKVIGRGAFGEVQLVRHKASQKVYAMKLLSKFEMIKRSDSAFFWEERDIMAFANSPW  151

ROCK1  136  VVQLFYAFQDDRYLYMVMEYMPGGDLVNLMSNYDVPEKWARFYTAEVVLALDAIHSMGFI  195
            VVQLFYAFQDDRYLYMVMEYMPGGDLVNLMSNYDVPEKWA+FYTAEVVLALDAIHSMG I
ROCK2  152  VVQLFYAFQDDRYLYMVMEYMPGGDLVNLMSNYDVPEKWAKFYTAEVVLALDAIHSMGLI  211

ROCK1  196  HRDVKPDNMLLDKSGHLKLADFGTCMKMNKEGMVRCDTAVGTPDYISPEVLKSQGGDGYY  255
            HRDVKPDNMLLDK GHLKLADFGTCMKM++ GMV CDTAVGTPDYISPEVLKSQGGDG+Y
ROCK2  212  HRDVKPDNMLLDKHGHLKLADFGTCMKMDETGMVHCDTAVGTPDYISPEVLKSQGGDGFY  271

ROCK1  256  GRECDWWSVGVFLYEMLVGDTPFYADSLVGTYSKIMNHKNSLTFPDDNDISKEAKNLICA  315
            GRECDWWSVGVFLYEMLVGDTPFYADSLVGTYSKIM+HKNSL FP+D +ISK AKNLICA
ROCK2  272  GRECDWWSVGVFLYEMLVGDTPFYADSLVGTYSKIMDHKNSLCFPEDAEISKHAKNLICA  331

ROCK1  316  FLTDREVRLGRNGVEEIKRHLFF  338
            FLTDREVRLGRNGVEEI++H FF
ROCK2  332  FLTDREVRLGRNGVEEIRQHPFF  354
```

(B)

NW Score	Identities	Positives	Gaps
1381	253/286 (88%)	271/286 (94%)	0/286 (0%)

```
CK2α    39   YQLVRKLGRGKYSEVFEAINITNNEKVVVKILKPVKKKKIKREIKILENLRGGPNIITLA  98
             YQLVRKLGRGKYSEVFEAINITNNE+VVVKILKPVKKKKIKRE+KILENLRGG NII L
CK2α'   40   YQLVRKLGRGKYSEVFEAINITNNERVVVKILKPVKKKKIKREVKILENLRGGTNIIKLI  99

CK2α    99   DIVKDPVSRTPALVFEHVNNTDFKQLYQTLTDYDIRFYMYEILKALDYCHSMGIMHRDVK  158
             D VKDPVS+TPALVFE++NNTDFKQLYQ LTD+DIRFYMYE+LKALDYCHS GIMHRDVK
CK2α′   100  DTVKDPVSKTPALVFEYINNTDFKQLYQILTDFDIRFYMYELLKALDYCHSKGIMHRDVK  159

CK2α    159  PHNVMIDHEHRKLRLIDWGLAEFYHPGQEYNVRVASRYFKGPELLVDYQMYDYSLDMWSL  218
             PHNVMIDH+ +KLRLIDWGLAEFYHP QEYNVRVASRYFKGPELLVDYQMYDYSLDMWSL
CK2α′   160  PHNVMIDHQQKKLRLIDWGLAEFYHPAQEYNVRVASRYFKGPELLVDYQMYDYSLDMWSL  219

CK2α    219  GCMLASMIFRKEPFFHGHDNYDQLVRIAKVLGTEDLYDYIDKYNIELDPRFNDILGRHSR  278
             GCMLASMIFR+EPFFHG DNYDQLVRIAKVLGTE+LY Y+ KY+I+LDP FNDILG+HSR
CK2α′   220  GCMLASMIFRREPFFHGQDNYDQLVRIAKVLGTEELYGYLKKYHIDLDPHFNDILGQHSR  279

CK2α    279  KRWERFVHSENQHLVSPEALDFLDKLLRYDHQSRLTAREAMEHPYF  324
             KRWE F+HSEN+HLVSPEALD LDKLLRYDHQ RLTA+EAMEHPYF
CK2α′   280  KRWENFIHSENRHLVSPEALDLLDKLLRYDHQQRLTAKEAMEHPYF  325
```

Figure S1. Pairwise alignment of kinase domains between ROCK1 and ROCK2 (A) and between CK2α and CK2α' (B). Protein sequences were aligned using NCBI BLAST global alignment tool (https://blast.ncbi.nlm.nih.gov/). NCBI protein ID: NP_005397.1 (ROCK1), NP_004841.2 (ROCK2), NP_001886.1 (CK2α), NP_001887.1 (CK2α').

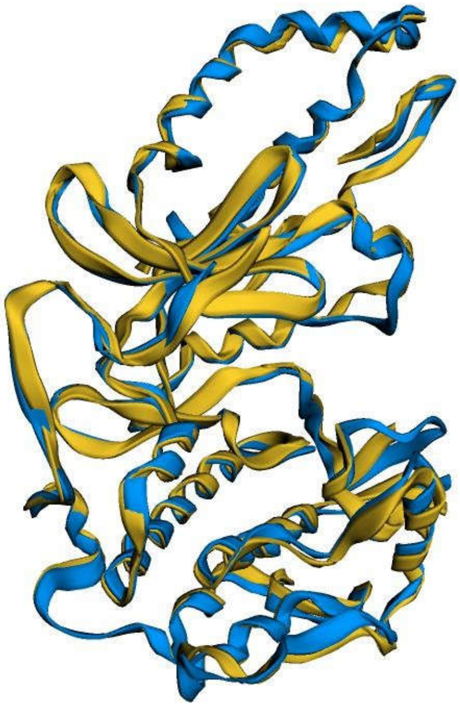

Figure S2. The structural alignment of ROCK1 and ROCK2. The structures were aligned using FATCAT program (https://fatcat.godziklab.org/). PDB IDs: 6E9W (ROCK1), 7JOV (ROCK2).
